# A non-enzymatic doxycycline absorbance sensor based on manganese-doped zinc sulfide nanoparticles coated with chitosan

**DOI:** 10.1371/journal.pone.0328304

**Published:** 2025-07-14

**Authors:** Son Hai Nguyen, Mai Thi Tran

**Affiliations:** 1 School of Mechanical Engineering, Hanoi University of Science and Technology, Hanoi, Vietnam; 2 College of Engineering and Computer Science, VinUniversity, Hanoi, Vietnam; 3 VinUni-Illinois Smart Health Center, VinUniversity, Hanoi, Vietnam; Hamadan University of Medical Sciences, IRAN, ISLAMIC REPUBLIC OF

## Abstract

The need for doxycycline detection is urgent because it is a vital antibiotic extensively utilized to combat bacterial diseases in humans and animals, and it is now at the forefront of the global battle against antibiotic resistance. Our research introduces an absorbance biosensor that employs chitosan-capped ZnS nanoparticles doped with manganese without an enzyme to address this critical need. Our study highlights the sensor’s robust performance with nearly 100% recovery and stability over time to detect doxycycline from 0 to 72.2 pM. Limits of detection and quantification were achieved at 4.5 pM and 15 pM, respectively, using simple absorbance measurements, with effective operation demonstrated across various media. The sensor also obtains excellent selectivity when testing with other analytes, including glucose, amoxicillin, tetracycline, penicillin, ampicillin, and cephalexin. The capabilities of these ZnS: Mn/Chitosan-based sensors mark a significant advancement in antibiotic monitoring, offering promising applications in clinical settings for patient care and environmental surveillance, thereby playing a critical role in curbing the spread of antibiotic resistance globally.

## Introduction

Antibiotics represent a cornerstone of modern medicine and are essential for treating many bacterial infections across clinical and veterinary settings. Among them, doxycycline (DOX), a tetracycline derivative, is especially valued for its efficacy against many bacterial species and its ability to treat diseases from acne to pneumonia and Lyme disease [1]. The extensive use of this antibiotic has inadvertently contributed to the rise and dissemination of antibiotic-resistant bacteria, presenting an escalating challenge to global health [2]. Hence, detecting DOX in both clinical and environmental samples has become a crucial area of focus in the battle against antibiotic resistance. Monitoring DOX levels can help track the distribution and concentration of this antibiotic, providing essential data that can be used to assess the risk of resistance development. Furthermore, as antibiotic resistance continues to evolve, enhancing our detection methods for DOX and other antibiotics is imperative for sustaining their effectiveness, safeguarding public health, and shaping future antibiotic policies [3].

Current antibiotic detection methods have significant limitations, including high costs, complex procedures, and lengthy processing times [4–7]. While techniques like high-performance liquid chromatography and mass spectrometry offer sensitivity and precision, they require expensive equipment and skilled operators. In contrast, microbiological assays are more affordable but lack specificity. These challenges highlight the need for alternative approaches, such as nanomaterial-based antibiotic sensors, which offer rapid, cost-effective, and highly sensitive detection with minimal sample preparation.

Researchers have successfully created a range of biosensors, including those based on electrochemical, optical, and field-effect transistor technologies [8–10]. Notably, optical biosensors stand out as they leverage nanomaterials’ unique optical properties and the precise selectivity of biorecognition elements to improve antibiotic detection. [11–15]. For example, enzyme absorbance biosensors employing zinc sulfide doped with manganese and coated with chitosan have demonstrated the ability to detect ampicillin from 13 to 72 μM, with a limit of detection (LOD) of 2.93 μM [16]. In another work, fluorescent sensors from graphene quantum dots with carboxyl groups have effectively detected tetracycline in milk, achieving a LOD of 8.2 nM [17]. Furthermore, a fluorescent aptasensor utilizing graphene oxide hydrogel has been designed to detect oxytetracycline within a range of 25–1000 μg/L, with a quantitation limit of 25 μg/L [18]. These studies illustrate the potential of nanomaterials in constructing highly sensitive and specific biosensors for monitoring antibiotics in various environments. Nonetheless, many documented approaches rely on enzymes or aptamers, which are susceptible to environmental changes, such as fluctuations in temperature and pH, leading to decreased stability [19]. There is a promising avenue in developing new nanomaterials that can bind directly to target molecules without these biorecognition agents, thereby circumventing stability issues [20]. This innovation has the potential to enable the creation of enzyme-free biosensors, offering a cost-effective solution while streamlining the preparation process.

Manganese-doped zinc sulfide coated with chitosan ((ZnS:Mn)@CH) materials are gaining attention as a promising platform for biosensor development aimed at antibiotic detection, thanks to their distinctive physicochemical properties and biocompatibility [21,22]. When Mn^2+^ ions are incorporated into the ZnS lattice, they serve as luminescent centers that facilitate efficient energy transfer from the ZnS host to the Mn^2+^ ions. This process enhances the emission intensity and stability, typically resulting in strong orange photoluminescence around 585–600 nm [23,24]. These photophysical improvements are crucial for biosensing applications, as a stronger and more stable emission provides higher sensitivity and lower detection limits for analytes. Moreover, the robust luminescent response of Mn-doped ZnS nanoparticles contributes to reliable and reproducible sensor performance, even in complex sample environments. The addition of a chitosan coating further facilitates dispersion, improves biocompatibility, and promotes interaction with target molecules, making (ZnS:Mn)@CH nanomaterials highly attractive for the development of sensitive and selective biosensors [25,26].

While chitosan-capped ZnS:Mn nanoparticles have previously shown promise in biosensor applications, the development of practical, accessible devices for antibiotic detection remains limited. Our prior work has primarily focused on advanced fluorescence-based detection methods, which, although highly sensitive, require specialized equipment and may not be suitable for widespread, routine monitoring [16,22,27]. In contrast, the present study pioneers the use of chitosan-capped ZnS:Mn nanoparticles for the absorbance-based detection of DOX, marking the first report of such an approach. By employing a simple UV-vis spectrophotometric readout, we offer a cost-effective, robust, and highly accessible biosensor platform. Importantly, our work demonstrates that this absorbance-based method achieves excellent sensitivity and selectivity for DOX, while also enabling broader adoption in field and resource-limited settings where fluorescence instrumentation is unavailable. Moreover, we systematically optimize the concentration of (ZnS:Mn)@CH nanomaterials to maximize sensing performance and evaluate sensor applicability across different media, thereby expanding the practical utility and versatility of the platform. Collectively, these advancements establish the current study as a significant contribution to the field, providing new analytical insights and practical strategies for antibiotic detection beyond those achieved in our previous fluorescence and tetracycline-based studies [16,22,28].

## Materials and methods

### Chemicals and synthesis of (ZnS:Mn)@CH nanomaterials

The chemicals and preparation process of sensing materials (ZnS:Mn)@CH were described in detail in [16,22]. Chitosan (CAS No. 9012-76-4) was purchased from Shanghai Zhanyun Chemical Co., Ltd. According to the product specification, the chitosan used in this study has a degree of deacetylation of ≥ 90% and a molecular weight of approximately 100–400 kDa. A high degree of deacetylation provides a greater density of free amino groups, improving solubility in acidic media and enhancing the availability of functional groups for interaction with target analytes or for further chemical modification [29]. The analytes used in this study include doxycycline hyclate (high purity, BioBasic, Ontario, Canada, Cat. No. 24390-14-5), tetracycline hydrochloride (TET, ultra-pure, BioBasic, Cat. No. 64-75-5), and D-glucose monohydrate (99.99%, BioBasic, Cat. No. 14431-43-7). Ampicillin sodium salt (AMP, 99.99%) was purchased from Njduly, China (Cat. No. 69-52-3). Penicillin G sodium (PCN, ultra-pure) was obtained from Bomeibio, China (Cat. No. 69-57-8). Amoxicillin crystalline (AMX, 99%, (Cat. No. 26787-78-0)) and cephalexin monohydrate (CEX, 98%, (Cat. No. 23325-78-2)) were acquired from Macklin, Shanghai, China. All chemicals were laboratory grade and used as received.

### Apparatus

All absorbance measurements were performed using a DeNovix DS-11 FX + UV-visible spectrophotometer. The spectrometer was operated in the absorbance mode with a wavelength range of 200–800 nm and a spectral bandwidth of 2 nm. Each spectrum was recorded under ambient laborator conditions using quartz cuvettes with a 1 cm path length.

### Solution preparation and measurement procedure

For each sensing experiment, (ZnS:Mn)@CH nanoparticle suspensions were prepared at a standard concentration of 500 mg/L in deionized (DI) water unless otherwise specified. Additional solutions with concentrations of 300, 400, 500, 600, and 700 mg/L were also prepared to evaluate the effect of material concentration. Each working solution was freshly prepared by dispersing the appropriate amount of nanomaterial in 10 mL of DI water and sonicating for 10 minutes to ensure homogeneity.

Analyte stock solutions of DOX were first prepared at 1 mM in DI water and serially diluted to obtain the working concentrations: 13.1, 33.3, 48.1, 59.4, 68.4, and 72.2 pM. For each test, 990 μL of the (ZnS:Mn)@CH suspension was transferred to a cuvette, and 10 μL of the analyte solution at the desired concentration was added, resulting in a final volume of 1 mL. The mixture was gently vortexed for 10 seconds to ensure complete mixing. Absorbance was then measured at specific time intervals (within 10 minutes after addition). Each concentration point was measured in three independent replicates, with three absorbance readings per replicate at 1-minute intervals.

To assess selectivity and matrix effects, the sensing protocol was systematically applied to milk, tap water, and bottled water by preparing analyte dilutions directly in each matrix. For every medium, a series of known DOX concentrations were spiked, and absorbance was measured under identical experimental conditions. Calibration curves at specific wavelengths were then constructed by plotting the changes in absorbance at these wavelengths versus DOX concentration in each respective medium. This systematic approach enabled both the optimization of sensor performance and a comprehensive evaluation of the sensor’s real-world applicability.

## Results and discussions

### Enzyme-free biosensors based on (ZnS:Mn)@CH to detect Doxycycline

**Fig 1:**
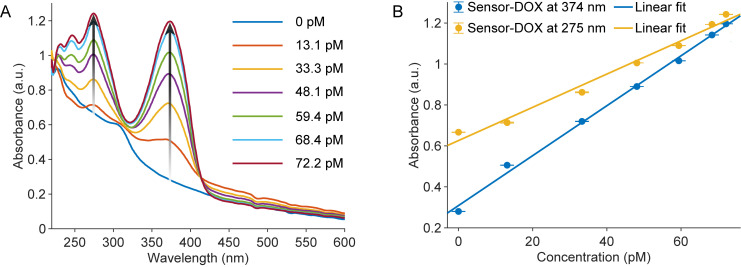
Absorbance measurement analysis. (A) Absorbance spectra of (ZnS:Mn)@CH-based sensors in contact with various DOX concentrations, (B) The relationship between sensor absorbances at 275 nm and 374 nm and DOX concentrations. Error bars indicate the standard deviation from nine measurements. Each data point (N = 9) reflects three independent replicates, with three measurements per replicate.


In previous work, ZnS:Mn nanoparticles coated with chitosan ((ZnS:Mn)CS) were successfully synthesized and thoroughly characterized using X-ray diffraction (XRD), Fourier-transform infrared spectroscopy (FTIR), and scanning electron microscopy (SEM) [16,22]. The nanocomposites exhibited a uniform nanoparticulate morphology, with average particle sizes around 50 nm and a net-like interconnected structure attributed to the chitosan coating. XRD analysis confirmed the preservation of the cubic sphalerite structure of ZnS upon Mn^2+^ doping, with characteristic peaks at the (111), (220), and (311) reflections, while FTIR spectra verified the incorporation of both chitosan and Mn^2+^ into the ZnS matrix. These findings collectively demonstrate the successful synthesis of (ZnS:Mn)CS nanocomposites. In this study, we present their application as a sensing platform for the non-enzyme detection of DOX in the range of 13.1 to 72.2 pM. These specific concentrations were selected based on the actual values obtained through serial dilution of stock solutions, which enabled us to evaluate sensor performance at the picomolar level. Due to the low concentrations involved, DOX exhibits negligible absorbance (S1 Fig). However, upon the addition of this antibiotic to the sensors, there was a significant enhancement in absorbance at 275 nm and 374 nm wavelengths (Fig 1A). In addition, the Sensor-DOX absorbance spectra displayed positive slopes corresponding to DOX concentrations at both 275 nm and 374 nm wavelengths (Fig 1B). The correlation between absorbance and DOX concentrations at these wavelengths can be expressed through the following linear equations:

$$A_{275}=\;0.0081C\;+\;0.626,\;\;\;\;(R^{2}\;=\;0.985)$$ (1)

$$A_{374}\;=\;0.0122C\;+\;0.309,\;\;\;\;(R^{2}=0.996)$$ (2)

where *C* represents the DOX concentration (pM), and A_275_ is the measured absorbance at 275 nm; A_374_ is the measured absorbance at 374 nm.

The LOD and limit of quantification (LOQ) for the sensor were determined based on the standard deviation of the blank (σ) and the slope of the calibration curve (S). Specifically, the LOD was calculated using the 3σ method (LOD = 3σ/S), where σ is the standard deviation of replicate blank measurements, and S is the slope of the corresponding calibration curve. The LOQ was calculated similarly, using the 10σ method (LOQ = 10σ/S). Based on these calculations, the LODs for Eqs (1) and (2) were 9.13 pM and 4.5 pM, respectively, while the LOQs were 30.4 pM and 15 pM.

In the validation phase to assess sensor performance, four samples with known DOX concentrations (24.05, 42.23, 54.11, and 64.13 pM) were prepared. For each spiked sample, absorbance was measured three times at 1-minute intervals using the same cuvette. The reported values in Table 1 represent the average of these three independent measurements. DOX concentrations were then calculated using Eqs (1) and (2). As shown in Table 1, the results indicate consistently high recovery rates, particularly for Eq (2) at 374 nm, which ranged from 94% to 105%. Based on these results, absorbance at 374 nm was selected for subsequent experiments.

**Table 1:** Validation of proposed sensors using the known concentration samples.

C_Spiked_ (pM)	275 nm			374 nm		
	A_Measured_ (a.u.)	C_Estimated_ (pM)	Recovery (%)	A_Measured_ (a.u.)	C_Estimated_ (pM)	Recovery (%)
24.05	0.79	20.62	86	0.62	25.22	105
41.23	0.94	38.86	94	0.81	40.69	99
54.11	1.02	49.11	91	0.93	50.66	94
64.13	1.11	59.38	93	1.06	61.34	96

C_Spiked_: the spiked concentration of a validated sample; A_Measured_: the absorbance of sensors measured at 275 or 374 nm; C_Estimated_ is the concentration calculated using equation (1) or (2).

**Fig 2:**
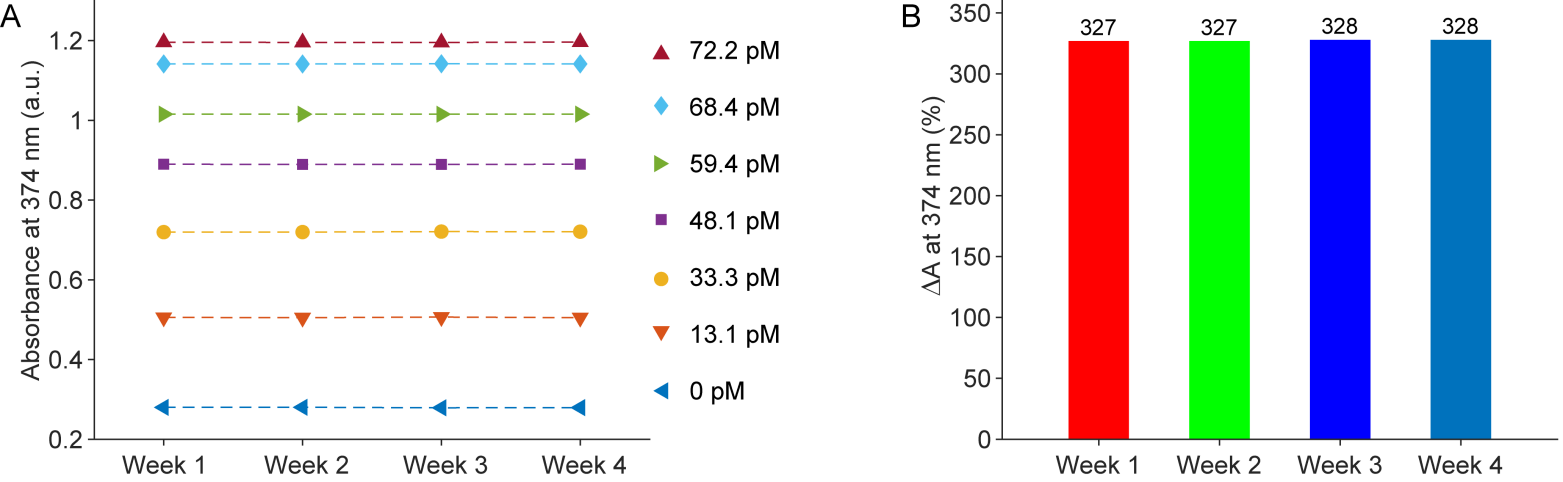
The stability of sensors after four weeks. (A) Absorbance measurements at 374 nm over the DOX concentrations. (B) Differential absorbance (ΔA) before and after adding 72.2 pM of DOX over the 4-week period.


The results thus far demonstrate the high sensitivity and reliability of the enzyme-free absorbance sensor. As a critical next step, we assessed the stability of the proposed sensors by conducting repeated experiments weekly over four weeks. Fig 2A illustrates the absorbance measurements at 374 nm in relation to the DOX concentrations, showing remarkably consistent values across the four weeks. Fig 2B displays the differential absorbance (ΔA) before and after adding 72.2 pM of DOX. Here, ΔA is defined as

$$\Delta A=\frac{A_{1}-A}{A}\times 100\;\%$$ (3)

where A and A_1_ are the absorbances before and after adding 72.2 pM DOX, respectively. The differences are nearly identical, at 327% and 328%, respectively. This exceptional consistency underscores the high stability of the proposed sensors, confirming their potential for reliable long-term use.

### Selectivity of absorbance-based sensors utilizing (ZnS:Mn)@CH nanomaterials

**Fig 3:**
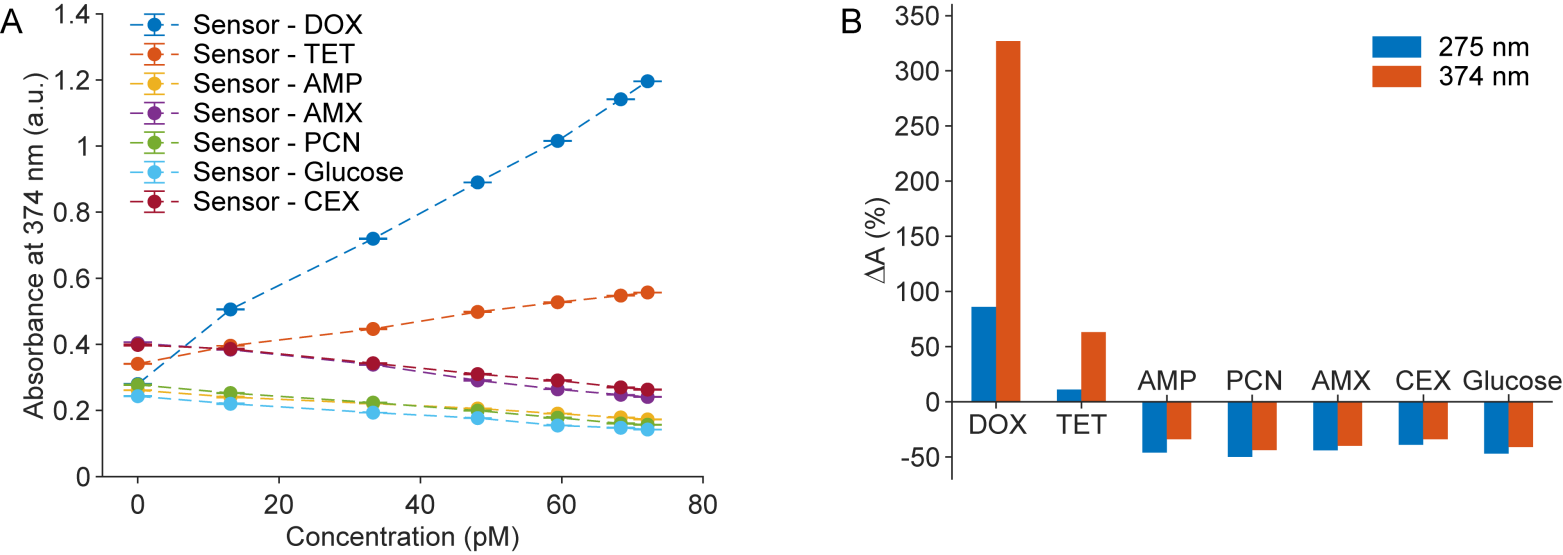
Selectivity of the proposed sensor. (A) Absorbance changes at 374 nm as a function of analyte concentration, (B) Percentage variation in absorbance at two wavelengths following sensor interaction with various analytes. Each data point (N = 9) reflects three independent replicates, with three measurements per replicate; error bars indicate standard deviation.

In this study, we evaluated the specificity of our sensors by testing them against common antibiotics, including AMX, TET, PCN, AMP, and CEX, along with the non-antibiotic compound glucose. The absorbance measurements from these tests, provided in S2 Fig, clearly differ from each other. Notably, as their concentrations increased, AMP, PCN, AMX, CEX, and glucose primarily exhibited a quenching effect. In contrast, TET, which belongs to the same antibiotic class as DOX, produced a mixed response: the sensor exhibited enhanced absorbance at 275 nm and 374 nm, but quenching effects at 250 nm and 320 nm (S2F Fig). The increases in absorbance observed for TET at 275 nm and 374 nm were substantially lower than those induced by DOX. To illustrate these findings, Fig 3A presents the absorbance changes at 374 nm as a function of analyte concentration, while Fig 3B compares absorbance differences before and after adding 72.2 pM of each analyte. DOX exhibited the highest positive slope and the most significant absorbance change, indicating the sensor’s high selectivity.

DOX, a tetracycline derivative, possesses various functional groups, such as a phenyl ring, hydroxyl groups, a tertiary amine, and carbonyl groups. The amino groups in chitosan enable hydrogen bonding with the oxygen and nitrogen atoms in DOX, promoting this interaction. Additionally, the hydroxyl group at the C6 position in TET may induce electrostatic repulsion, diminishing its interactions with (ZnS:Mn)@CH. Unlike TET, DOX does not have a hydroxyl group at the C6 position, which enhances its likelihood of effective interactions with (ZnS:Mn)@CH, resulting in superior selectivity in detecting DOX [27].

**Fig 4:**
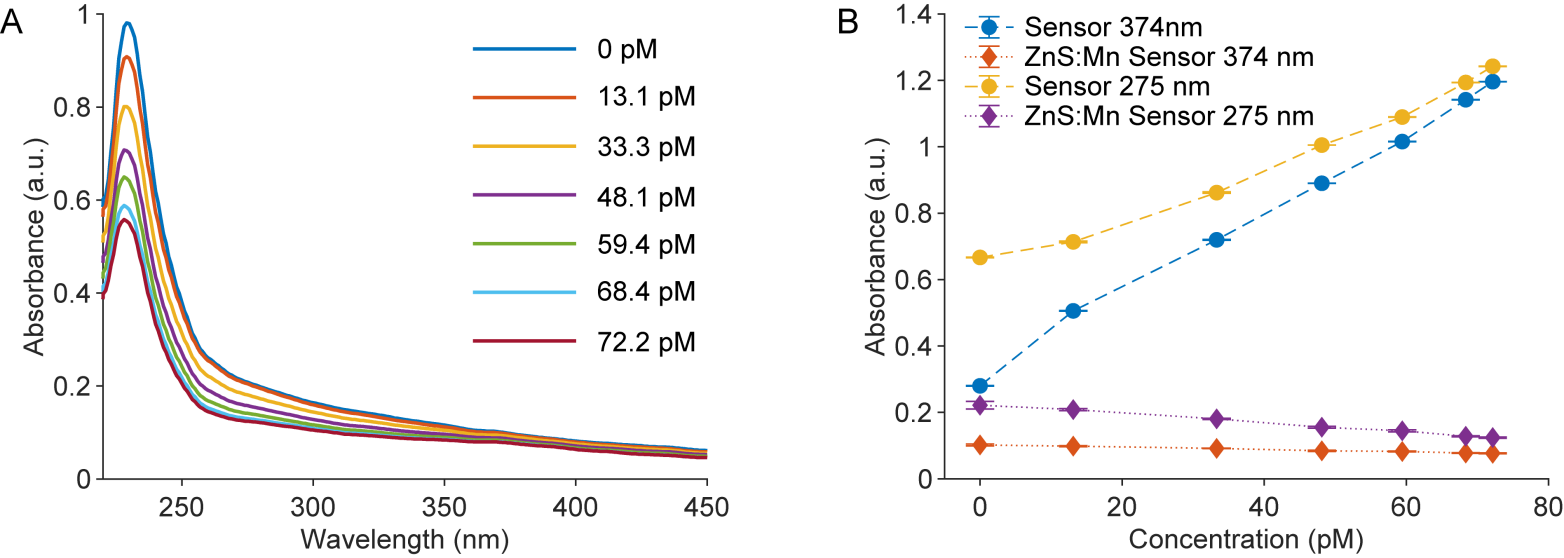
Performance of (ZnS:Mn)-based sensors. (A) Absorbance spectra of (ZnS:Mn)-based sensors upon interaction with varying DOX concentrations. (B) Absorbance variations at two wavelengths in response to different DOX levels for both sensor types. Each data point (N = 9) reflects three independent replicates, with three measurements per replicate; error bars indicate standard deviation.

The enhanced selectivity of the sensor is primarily attributed to its material structure, which notably omits the use of conventional bioreceptors. This design significantly influences the sensor’s ability to selectively distinguish between different analytes. In particular, the platform demonstrates outstanding selectivity for DOX detection without relying on enzymatic activity, suggesting that the chitosan layer plays a pivotal role in this selective recognition. To test this hypothesis, control experiments were performed using nanoparticles synthesized using the same protocol, except that chitosan was omitted during preparation. The reference material (ZnS:Mn) is detailed in S3 Fig. The absorbance spectra of the reference sensors that consist of (ZnS:Mn) in contact with DOX are displayed in Fig 4A. Without the chitosan layer, only a quenching effect was observed within the testing wavelength range. The absorbance values at 275 nm and 374 nm for the reference sensors were plotted alongside those of the (ZnS:Mn)@CH sensors in Fig 4B to underscore the contrasting behaviors of both sensors. This distinction further highlights chitosan’s crucial role in DOX detection, providing a reactive surface that enhances DOX binding through electrostatic attraction, hydrogen bonding, or possible covalent interactions.

To quantitatively assess the significance of these differences, independent t-tests were performed on the absorbance values at both 275 nm and 374 nm. The (ZnS:Mn)@CH sensors exhibited markedly higher absorbance responses at both wavelengths compared to the control sensors prepared without chitosan. Specifically, at 275 nm, the mean absorbance for (ZnS:Mn)@CH sensors was 0.97 ± 0.23, while the control group showed a mean of only 0.17 ± 0.04 (p = 0.000067). At 374 nm, the (ZnS:Mn)@CH sensors had a mean absorbance of 0.82 ± 0.34, compared to 0.09 ± 0.01 for the controls (p = 0.0012). These highly significant differences statistically confirm that the presence of chitosan is essential for the enhanced sensitivity and selectivity of DOX detection observed in our system.

### The optimal concentration of sensing materials and the feasibility of proposed sensors in various working media

During the initial phase of biosensor development for DOX detection, sensors were fabricated with different (ZnS:Mn)@CH concentrations, ranging from 300 mg/L to 700 mg/L. As shown in S4 Fig, the absorbance spectra of these sensors, both before and after exposure to DOX, revealed that the overall absorbance increased proportionally with higher nanoparticle concentrations. This observation is explained by the intrinsic light-absorbing properties of (ZnS:Mn)@CH nanoparticles; as their concentration increases, more light is absorbed at specific wavelengths, consistent with the Beer-Lambert law. Across all concentrations tested, the sensors displayed consistent response behaviors, with two prominent absorbance peaks at 275 nm and 374 nm. S1 Table further summarizes the absorbance variations at these wavelengths in response to different DOX concentrations for each level of sensing material.

**Fig 5:**
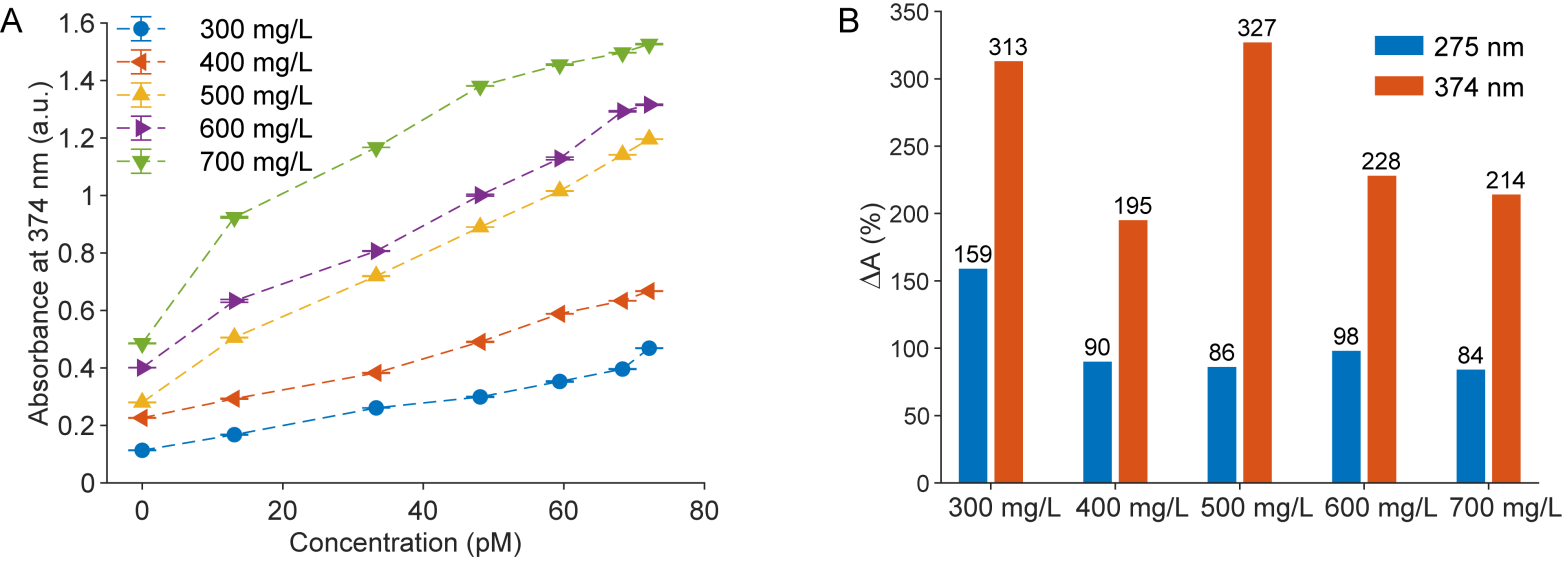
Dependency of sensor performance on the concentration of sensing materials. (A) The absorbances at 374 nm of five sensors change with the DOX concentration. Each data point (N = 9) reflects three independent replicates, with three measurements per replicate; error bars indicate standard deviation. (B) Percentages of absorbance change of five sensors after adding 72.2 pM DOX.

Additional insights are provided in Fig 5A, which shows the absorbance changes at 374 nm in response to increasing DOX concentrations, while Fig 5B highlights the absorbance change before and after the addition of 72.2 pM DOX. In S1 Table, comprehensive calibration data for all tested (ZnS:Mn)@CH concentrations are summarized, including slopes and correlation coefficients (R^2^). The sensor containing 500 mg/L of (ZnS:Mn)@CH demonstrated the highest overall effectiveness, with steep calibration slopes (0.0081 at 275 nm and 0.0122 at 374 nm), strong linear correlations (R^2^ = 0.985 and 0.996, respectively), and the largest absolute change in absorbance at 374 nm (327%). Although slightly higher slopes were observed at higher concentrations, these were accompanied by reduced linearity and diminishing improvement in absorbance response. Collectively, these metrics confirm that 500 mg/L provides an optimal balance of sensitivity, linearity, and signal response, supporting its selection as the optimal concentration for sensor design in this study.

**Fig 6:**
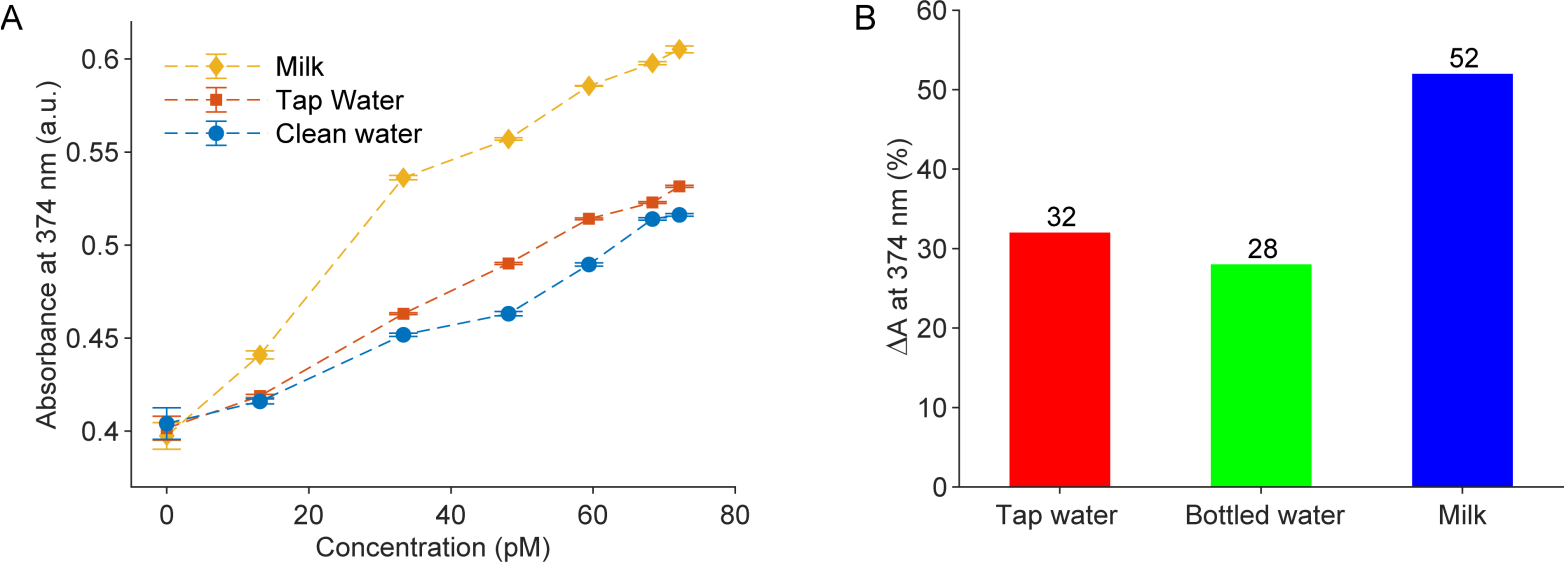
Sensors in various media. (A) The absorbances at 374 nm of the proposed sensors change with the DOX concentrations in three working environments, including bottled water, tap water, and milk. Each data point (N = 9) reflects three independent replicates, with three measurements per replicate; error bars indicate standard deviation. (B) percentages of absorbance change of three tests after adding 72.2 pM DOX.

The optimized sensors were tested with DOX spiked into different media, including bottled water, tap water, and milk, to evaluate sensor performance across a range of real-world sample matrices. Bottled water and tap water represent common environmental sources where antibiotic contamination may occur, while milk was selected due to the widespread use of doxycycline in veterinary medicine for dairy animals and the importance of monitoring antibiotic residues in food products for consumer safety. S5 Fig presents the absorbance spectra of the sensors upon DOX addition in these environments, consistently showing enhancement effects at 275 nm and 374 nm. Fig 6A illustrates the absorbance variations at 374 nm as a function of DOX concentration, while Fig 6B shows absorbance changes before and after introducing 72.2 pM DOX. Across all tests, the absorbance changes followed linear trends, as summarized in Table 2.

**Table 2:** The fitting equations and limit of detection of sensors with DOX spiked in different media.

Medium	Absorbance at 374 nm	R^2^	Detection limit (pM)
Bottled water	0.0016*C *+ 0.3981	0.979	10.64
Tap water	0.0019*C *+ 0.3996	0.996	4.77
Milk	0.0029*C* + 0.4106	0.963	14.2

*C* represents the DOX concentration (pM).

Remarkably, the sensors exhibited the highest sensitivity in milk, as indicated by the steepest calibration slope (0.0029), a 52% change in absorbance at 374 nm after adding 72.2 pM DOX, and a detection limit of 14.2 pM (Fig 6, Table 2). This enhanced response may be due to interactions between DOX and milk constituents, such as proteins or calcium ions, which could facilitate greater adsorption of DOX onto the sensor surface.

In contrast, sensor performance in tap and bottled water was notably lower, as reflected by lower calibration slopes (0.0019 and 0.0016, respectively), smaller changes in absorbance at 374 nm (32% for tap water and 28% for bottled water), and detection limits of 4.77 pM (tap) and 10.64 pM (bottled). These results suggest that the matrix composition, particularly the lower ionic strength and absence of organic or macromolecular content in water, reduces DOX–sensor interactions. Furthermore, even though the measured pH values for tap water (7.56), bottled water (7.24), and milk (7.2) are relatively similar, minor pH variations could also influence the charge states of the sensor and DOX, potentially impacting binding efficiency. The presence of trace metal ions or disinfectant residues in tap water may further contribute to reduced sensor performance. Overall, the highest sensor response in milk highlights the important role of sample matrix effects on analytical sensitivity and practical detection limits.

For all matrices, experiments were performed in three independent replicates, with three absorbance measurements per replicate at 1-minute intervals (N = 9 per data point), and the sensor response was recorded within 10 minutes of DOX addition. Time-course studies confirmed that absorbance signals remained stable, supporting the reliability of the sensor for practical use.

The biosensors based on (ZnS:Mn)@CH exhibit high sensitivity, selectivity, and stability in detecting DOX, providing a simple platform with straightforward preparation and measurement. This sensing technology surpasses previous methods due to its simplicity, cost-effectiveness, ultra-sensitive detection range, and lower detection limits. For instance, a prior study reported DOX sensors capable of detecting femtomolar concentrations using the same materials; however, it employed fluorescence-based detection, which required more sophisticated instrumentation [27]. Another study developed DOX sensors using boron- and nitrogen-codoped carbon quantum dots encapsulated in metal-organic frameworks combined with molecularly imprinted polymers, achieving a detection range of 0.05–20 mg/L with a LOD of 14.21 ng/mL [30]. Additionally, another fluorescence-based sensor utilizing a zinc coordination polymer detected DOX in the 0–30 µM range, with a LOD of 3.7 nM [31]. However, its detection range was significantly higher than the proposed sensor’s, and fluorescence techniques generally require more expensive instrumentation than the absorbance-based method used in this study. Moreover, the sensing materials in those sensors were more complex to prepare, and their detection range and LOD were more significant than those of the proposed sensor.

While (ZnS:Mn)@CH-based biosensors demonstrate high sensitivity and selectivity for DOX, several limitations remain. Most notably, we cannot exclude the possibility of nonspecific responses to other small molecules or structurally related antibiotics not included in our current testing panel. Further studies are needed to evaluate selectivity against a broader range of potential interferents, especially those found in real samples. Additionally, although the absorbance-based platform is cost-effective and accessible, its performance may be affected by sample matrix components, such as proteins or ions, that could cause signal variation or interference. Long-term operational stability and reproducibility under diverse real-world conditions also require further validation.

The following steps involve optimizing the working conditions and material synthesis for the proposed sensors to test DOX in actual samples. These advancements will expand the application of (ZnS:Mn)@CH nanomaterials to real-world scenarios, such as environmental monitoring, enabling on-site detection of antibiotic residues in water sources and food safety screening, for example, rapid testing of milk or dairy products for doxycycline contamination prior to distribution. Furthermore, this strategy lays the foundation for developing next-generation, enzyme-free antibiotic sensors leveraging nanomaterials for practical use in regulatory compliance, public health protection, and potentially clinical diagnostics. Future work should focus on expanding interference studies, optimizing sensor protocols for practical field deployment, and integrating the platform with portable readout systems. Addressing these challenges will be essential for advancing this sensor toward reliable use in environmental, food safety, or clinical applications.

## Conclusion

This study highlights the effectiveness of (ZnS:Mn)@CH biosensors in detecting DOX with outstanding sensitivity, selectivity, and stability over a range of 0 to 72.2 pM, achieving a detection limit of 4.5 pM. The sensors’ simplicity and cost-effectiveness present a significant advantage over more complex and costly existing technologies. The enzyme-free design reduces biotic interference and enhances stability under diverse conditions. They exhibit consistent performance in various media, including bottled and tap water and milk, making them versatile tools for food safety, environmental monitoring, and clinical diagnostics. This study fosters the advancement of nanomaterial-based biosensors, offering innovative solutions to address antibiotic contamination and resistance, thereby promoting safer food production, environmental protection, and public health.

## Supporting information

S1 Fig The absorbance spectra of different DOX concentrations. (TIF)

S2 Fig The absorbance spectra of sensors in contact with different analytes. (TIF)

S3 Fig Characterization of the reference material (ZnS:Mn). (A) SEM image; (B) XRD pattern; (C) FTIR spectrum.
(TIF)

S4 Fig Absorbance spectra of sensors before and after adding DOX. (A) Absorbance spectra of five sensors with different (ZnS:Mn)@CH concentrations before contact with DOX; (B, C, D, E, F) Absorbance spectra of sensor with 300 mg/L, 400 mg/L, 500 mg/L, 600 mg/L, 700 mg/L (ZnS:Mn)@CH contact with different DOX concentrations.
(TIF)

S5 Fig Potential of (ZnS:Mn)@CH-based sensors for DOX detection in various media. (TIF)

S1 Table Calibration data for all tested (ZnS:Mn)@CH concentrations. (PDF)

S1 File Raw data. (XLSX)
