# Supplementary figures and images for: A non-enzymatic doxycycline absorbance sensor based on manganese-doped zinc sulfide nanoparticles coated with chitosan
Source: PLoS One. 2025 Jul 14;20(7):e0328304. doi: 10.1371/journal.pone.0328304 (PMC12258549; doi:10.1371/journal.pone.0328304)

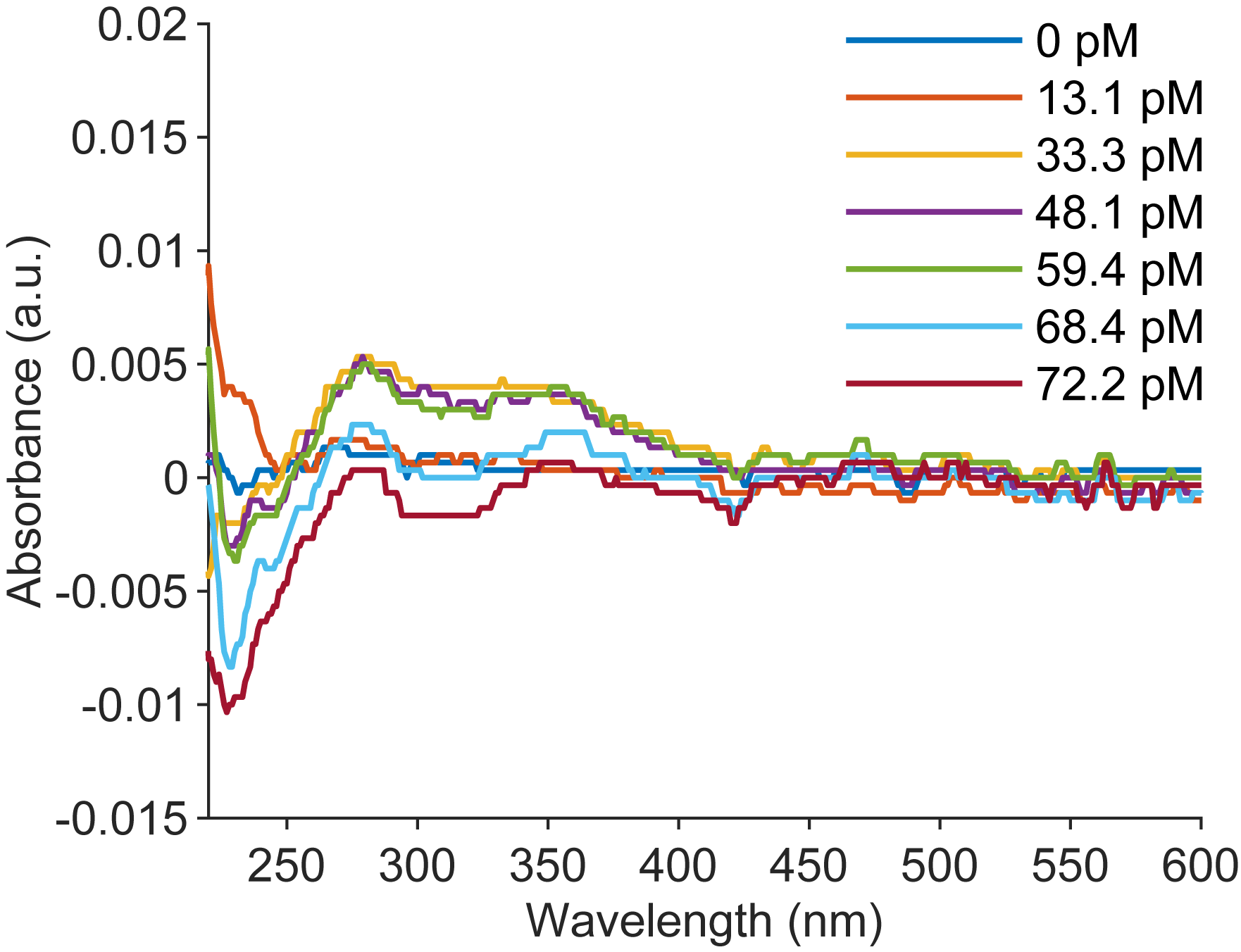

Supplement: S1 Fig — (TIF) [file pone.0328304.s001.tif]

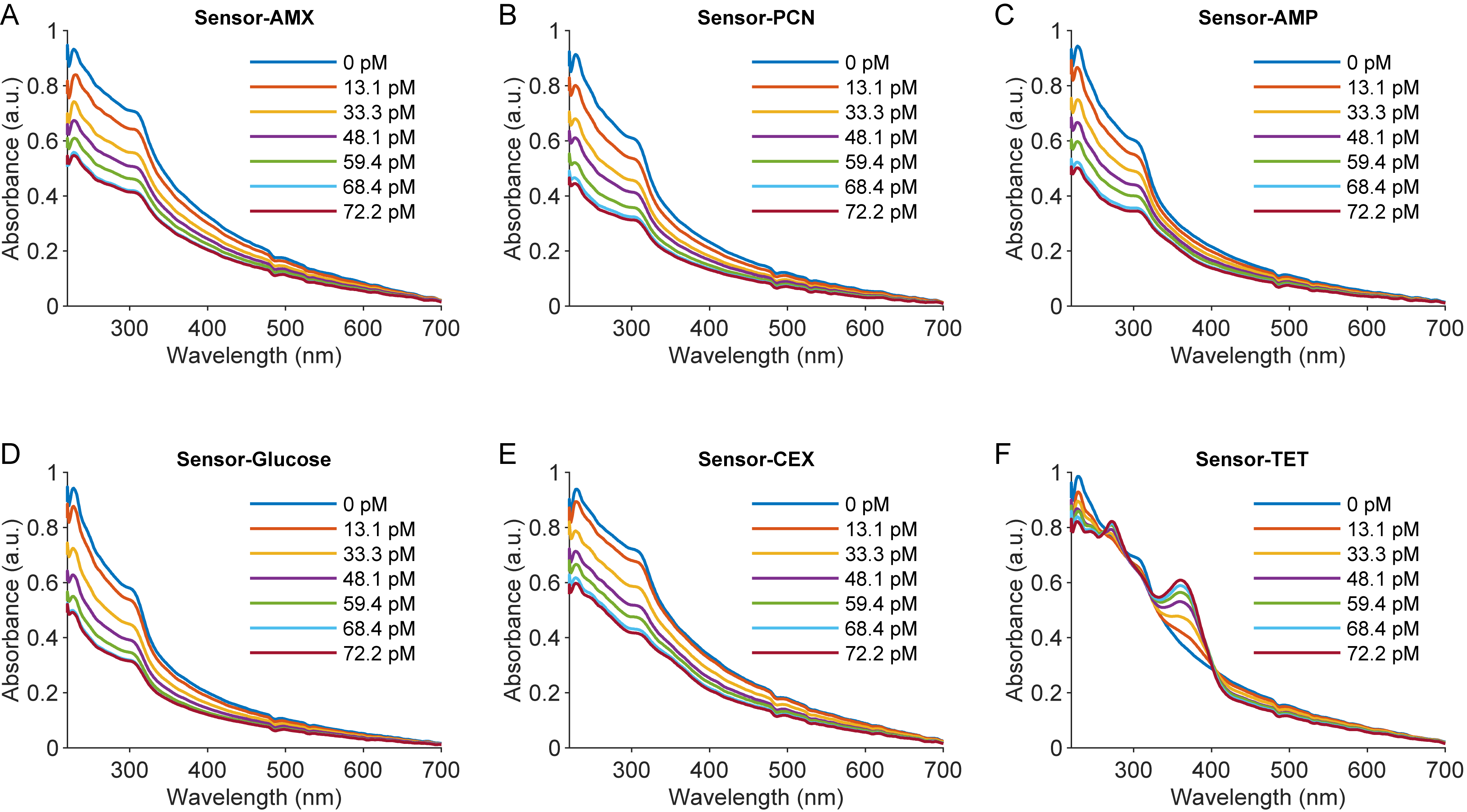

Supplement: S2 Fig — (TIF) [file pone.0328304.s002.tif]

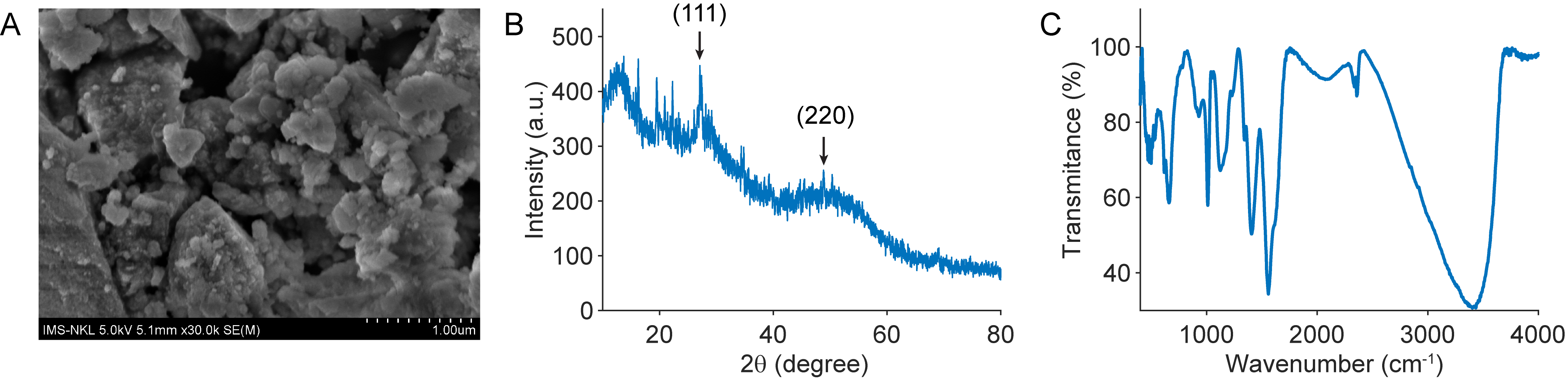

Supplement: S3 Fig — (A) SEM image; (B) XRD pattern; (C) FTIR spectrum. (TIF) [file pone.0328304.s003.tif]

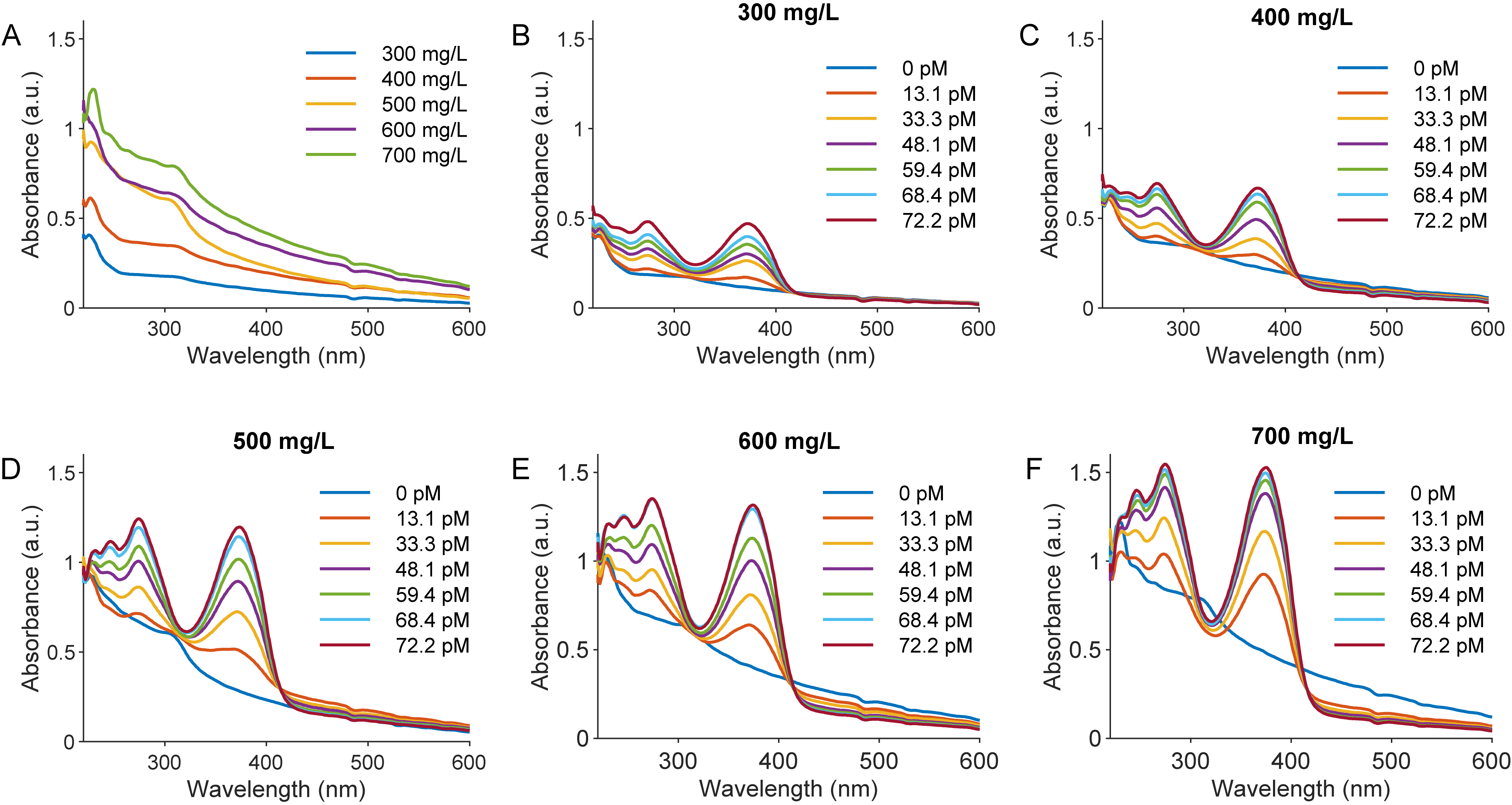

Supplement: S4 Fig — (A) Absorbance spectra of five sensors with different (ZnS:Mn)@CH concentrations before contact with DOX; (B, C, D, E, F) Absorbance spectra of sensor with 300 mg/L, 400 mg/L, 500 mg/L, 600 mg/L, 700 mg/L (ZnS:Mn)@CH contact with different DOX concentrations. (TIF) [file pone.0328304.s004.tif]

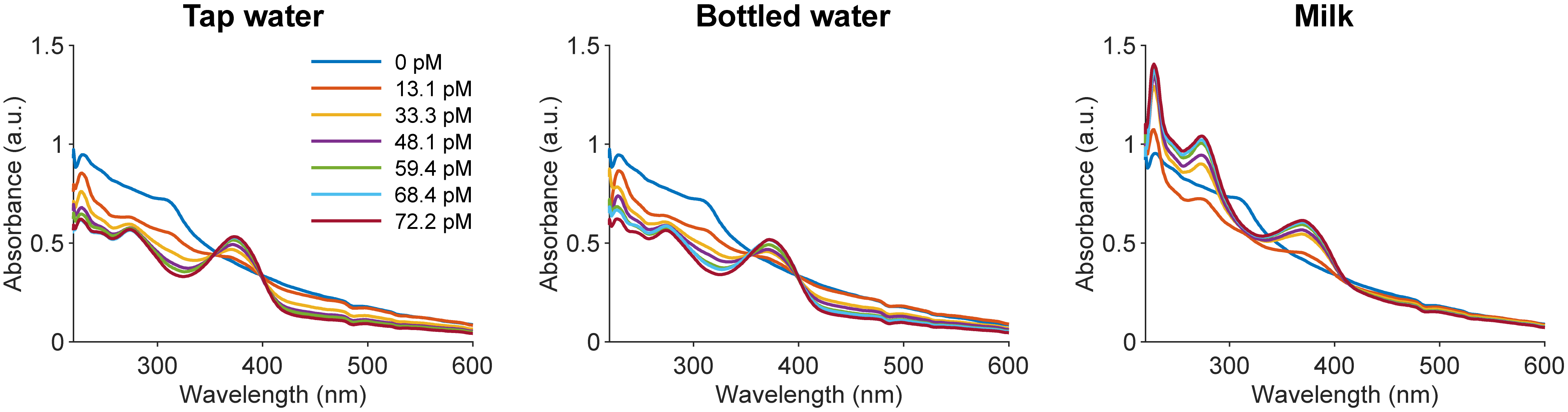

Supplement: S5 Fig — (TIF) [file pone.0328304.s005.tif]
