# Supplementary material for: A non-enzymatic doxycycline absorbance sensor based on manganese-doped zinc sulfide nanoparticles coated with chitosan
Source: PLoS One. 2025 Jul 14;20(7):e0328304. doi: 10.1371/journal.pone.0328304 (PMC12258549; doi:10.1371/journal.pone.0328304)
Supplement: S1 Table — (PDF) [file pone.0328304.s006.pdf]

S1 Table. Calibration data for all tested (ZnS:Mn)@CH concentrations.

(ZnS:Mn)@CH concentration (mg/L)	Absorbance at 275 nm	R^2	Absorbance at 374 nm	R^2
300	$0.0037C + 0.1718$	0.954	$0.0044C + 0.1071$	0.97
400	$0.0047C + 0.3428$	0.984	$0.0062C + 0.2087$	0.996
500	$0.0081C + 0.626$	0.985	$0.0122C + 0.3087$	0.996
600	$0.0092C + 0.6779$	0.986	$0.0123C + 0.4212$	0.993
700	$0.0096C + 0.892$	0.975	$0.0133C + 0.6451$	0.93

C represents the DOX concentration (pM).
